# aYChr-DB: a database of ancient human Y haplogroups

**DOI:** 10.1093/nargab/lqaa081

**Published:** 2020-10-09

**Authors:** Laurence Freeman, Conrad Stephen Brimacombe, Eran Elhaik

**Affiliations:** University of Sheffield, Department of Animal and Plant Sciences, Sheffield S10 2TN, UK; University of Sheffield, Department of Animal and Plant Sciences, Sheffield S10 2TN, UK; University of Bristol, Department of Archaeology and Anthropology, Bristol BS8 1TH, UK; University of Sheffield, Department of Animal and Plant Sciences, Sheffield S10 2TN, UK; Lund University, Department of Biology, Lund 223 62, Sweden

## Abstract

Ancient Y-Chromosomal DNA is an invaluable tool for dating and discerning the origins of migration routes and demographic processes that occurred thousands of years ago. Driven by the adoption of high-throughput sequencing and capture enrichment methods in paleogenomics, the number of published ancient genomes has nearly quadrupled within the last three years (2018–2020). Whereas ancient mtDNA haplogroup repositories are available, no similar resource exists for ancient Y-Chromosomal haplogroups. Here, we present aYChr-DB—a comprehensive collection of 1797 ancient Eurasian human Y-Chromosome haplogroups ranging from 44 930 BC to 1945 AD. We include descriptors of age, location, genomic coverage and associated archaeological cultures. We also produced a visualization of ancient Y haplogroup distribution over time. The aYChr-DB database is a valuable resource for population genomic and paleogenomic studies.

## INTRODUCTION

The genomic history of populations is a tapestry of undirected changes as no population remains immutable over time. Whereas coalescent and other reconstruction methods that rely on modern populations are inaccurate and carry a high risk of misinterpretation (1), analyzing the DNA of ancient human populations allows capturing their fine-scale population structure (2) and past events as they were. Combining this evidence with environmental, cultural and other genomic information enables a more accurate representation of the past (3).

The Y-Chromosome contains the largest nonrecombining block in the human genome (4). Using both traditional methods (e.g. PCR) and high-throughput sequencing, haplogroups of ancient individuals are identifiable, facilitating the study of past genetic diversity (3). Combining Y-DNA with radiocarbon dating also provides a means to map Y-chromosomes onto a phylogenetic tree, which can be used to assess whether previous reports of ancestral variation based on modern DNA are supported by ancient samples and if we can find representatives of ancient clades that are rare (5) or no longer exist (6).

Over the past 2 years, ancient Y-chromosomal data have begun to accumulate rapidly. Published data from the period 2007 to 2017 (480 Y chromosomes) was nearly quadrupled within the next 3 years 2018–2020 (1797 Y chromosomes) (Supplementary Table S1). In concert with mitochondrial DNA, Y-Chromosomal DNA has been used to study the origins of present-day and ancient Eurasians (7) along with their languages (8–11) and disease prevalence (3).

Only a handful of ancient DNA databases have been compiled to date, such as the Online Ancient Genome Repository (https://www.oagr.org.au), which primarily stores samples sequenced by the Australian Centre for Ancient DNA, and the AmtDB (12), which predominantly features ancient mtDNA. The lack of a dedicated database focusing on the collection of ancient Y-Chromosomal data has impeded research in the field and prompted us to develop aYChr-DB.

aYChr-DB collates a large proportion of the published Eurasian ancient Y-DNA data over the past 13 years (2007–2020) into an easily accessible archive. The manually curated database not only standardizes the reporting of data and makes haplogroup comparison feasible but also offers socio-cultural annotation. The genomic sequences are available through the source studies.

## MATERIALS AND METHODS

Relevant papers were identified by querying PubMed and Google Scholar with the key words ‘ancient Y’, ‘ancient haplogroup’ and ‘ancient DNA’ + ‘Y chromosome’. Both reviews and research articles were selected, with no restrictions on date of publication or journal of publication. Records were then manually curated to remove duplications.

Maps were drawn using the ggmap R package (13). aYChr-DB (Supplementary Table S1) is publicly and freely accessible at https://github.com/eelhaik/aYDB.

## RESULTS

aYChr-DB contains 1797 samples (Supplementary Table S1). Multiple descriptors are available for each sample, which are named according to the official/published ID, such as country and location. The age of the sample, where applicable, is provided in both BC and BP calibrated from 1950. Carbon-dated samples are shown as calBC/BP. For samples without published coordinate data, we provide coordinates based on location names and descriptions. The archaeological period of each sample has been assigned based on age and location. Where given, average genomic coverage has been included. The comments section clarifies additional information on the samples which may be pertinent to database users.

**Figure 1.**
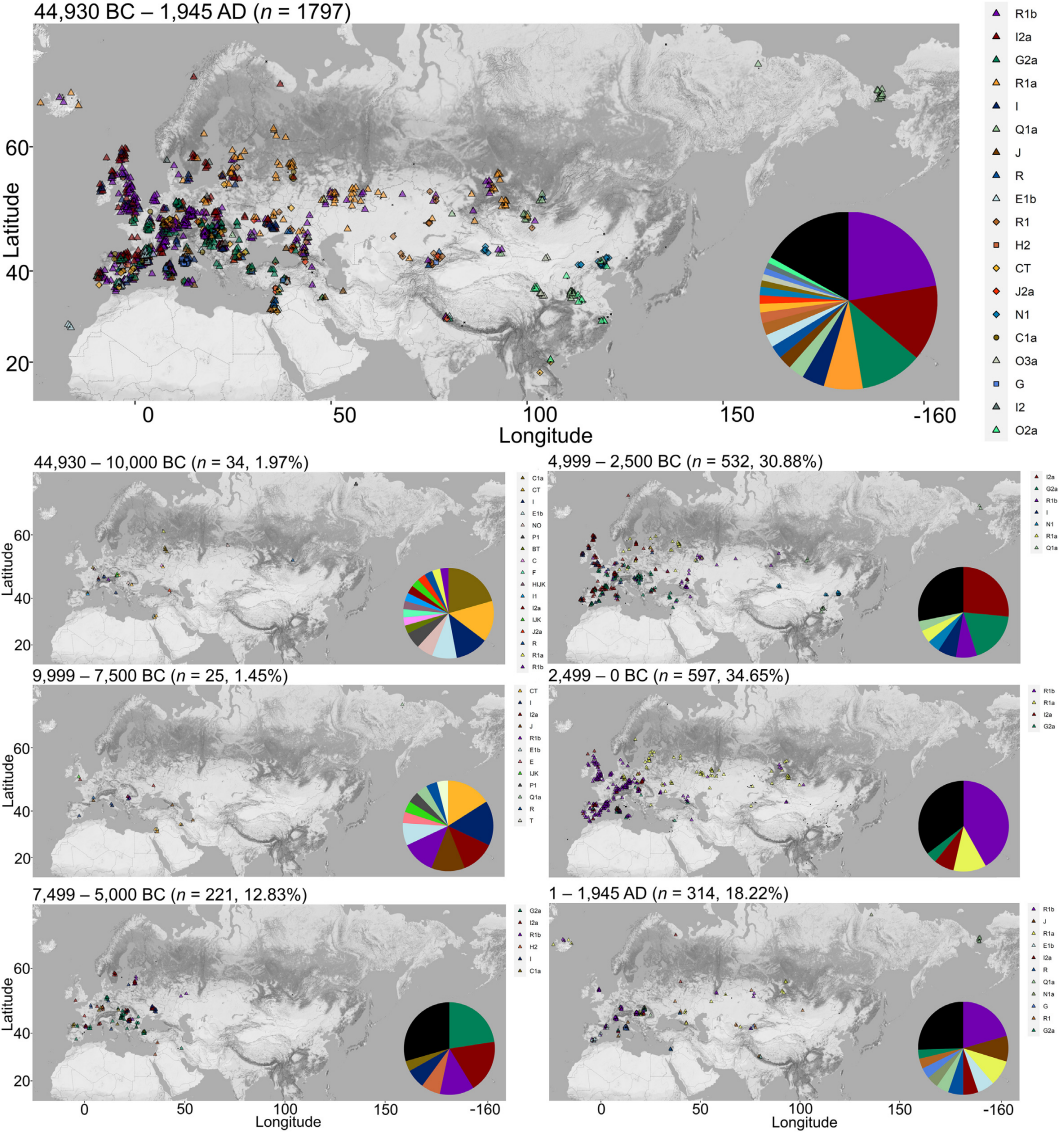
The geographical distribution of 1723 ancient Eurasian haplogroups over time. The location of each archaeological site is marked as a dot. Colored shapes denote the different haplogroups found on the site. A small random variation was used in the plotting to avoid cluttering. Low-frequency haplogroups (<3% in interval maps, <1% in ‘all time periods’ map) are represented as black wedges in the pie charts and their corresponding locations marked as black crosses on the maps.

We produced a visualization of the aYChr-DB—for a total of 1723 samples after removing 74 undated samples (Figure 1 and Supplementary Figure S1). The full 1797 samples were included in the main ‘all time periods’ map. For coherency, haplogroups were trimmed to three letters at most, (i.e. R1a1a1 is shown as R1a). Samples were classified into one of six periods, spanning the range of published dates, using the age or average age of the sample. Several trends are noteworthy. A large proportion (65.5%) of collected ancient samples are dated between 0 and 4999 BC. R1b is the modal haplogroup in the ancient Eurasian samples, accounting for 22.3% of the data. I2a is the second most common at 13.9%, followed by G2a at 11.3% and R1a at 7.1%. That the majority of the samples are located in Europe is likely due to the availability of large depositories and history of archaeological research in this region and its propensity for cool, temperate conditions suitable for the preservation of ancient DNA (14). Over 40% of the samples were found in four countries: Spain (11.4%), Russia (10.4%), Hungary (9.7%) and Italy (9.6%).

The major challenge in our efforts to provide coherent and useful annotation was in ascribing meaningful cultural information to the samples. European prehistoric periods are conventionally defined by technological innovations, excepting the Paleolithic-Mesolithic transition, which is a climate transition. The primary European cultural phases are the Neolithic, Copper Age, Bronze Age and Iron Age, followed by historic periods such as the Romans and Medieval periods. Up to the Bronze Age within Europe and West Asia, this technological framework is useful for geneticists as it often corresponds well with major shifts in population structure because these technologies enabled certain groups to move into adjacent regions. The Iron Age and beyond are characterized by advanced civilizations across Europe and West Asia, while in the colder and less fertile regions of Central and Northeastern Asia, nomadic, and hunter-gatherer lifestyles persisted in a scattering of small populations across a broad expanse of territory (15). These people often possessed iron and bronze technologies but had no sedentary agricultural base and demonstrated high mobility. Their cultures have been challenging to classify archaeologically in terms of any overarching technological or historical framework.

In East Asia, we can observe a parallel, although typically not synchronous development of agriculture, copper/bronze technology and eventually iron (16). The transition to agriculture does correspond with population movement (17,18) and is a pattern demonstrated throughout the region. However, subsequent archaeological transitions are usually referred to through dynastic change rather than technological change (19). This is particularly true within China and adjacent regions, despite migration associated with these technological shifts proven at a genetic level (18).

## DISCUSSION

We developed a database of ancient Eurasian Y-Chromosomal haplogroups, collating published data from the last 12 years. We assigned missing descriptors to many samples and provided a socio-cultural annotation, which contributes to the uniqueness and usefulness of this resource. Finally, a geographical visualization of the data provides a convenient review of the samples at discrete intervals.

Version 1.0 of the database includes samples from across Eurasia due to the rarity of ancient Y haplogroups from elsewhere. The database will be updated periodically with recently published Y-Chromosome data. We expect that later updates will provide a denser and more extensive global coverage of published data. We hope that the aYChr-DB will increase the accessibility and availability of ancient Y-DNA data.

## Supplementary Material

lqaa081_Supplemental_File
