# Supplementary material for: aYChr-DB: a database of ancient human Y haplogroups
Source: NAR Genom Bioinform. 2020 Oct 9;2(4):lqaa081. doi: 10.1093/nargab/lqaa081 (PMC7671346; doi:10.1093/nargab/lqaa081)

Supplementary information

Table S1

The ancient human Y haplogroup database. The database provides for each sample its age, country, site location, geographical region, geographical coordinates, given and revised periods, culture name, haplogroup, the type of haplogroup SNPs provided in the publication, coverage, and the reference.

This table is available via https://github.com/eelhaik/aYDB

Figure S1

The geographical distribution of the sites for the 1797 ancient Eurasian haplogroups over time. The location of each archaeological site is marked as a red dot.

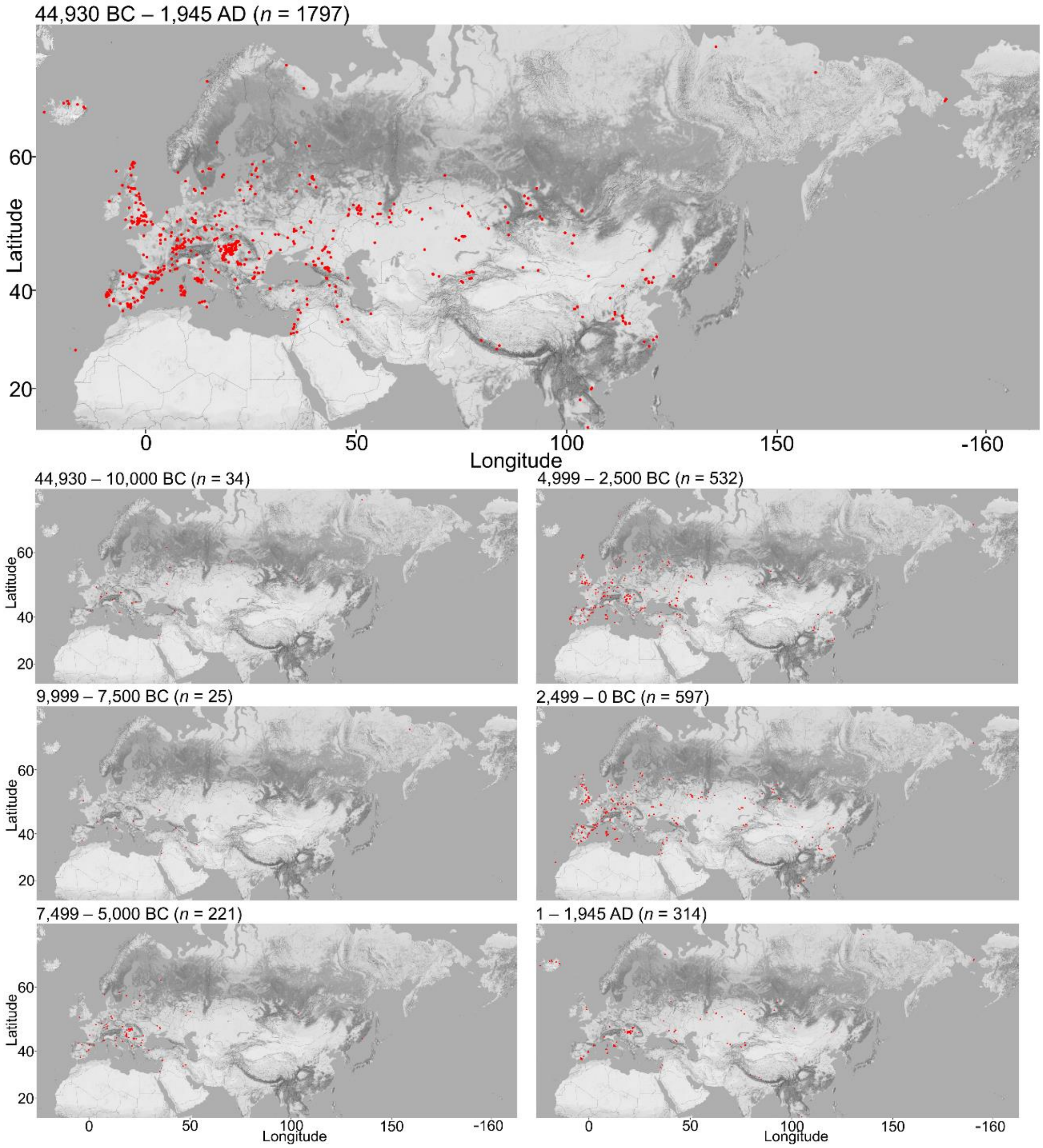

Supplement: lqaa081_Supplemental_File [file lqaa081_supplemental_file.pdf]
